# Effects of low-intensity ultrasound opening the blood-brain barrier on Alzheimer's disease—a mini review

**DOI:** 10.3389/fneur.2023.1274642

**Published:** 2023-11-01

**Authors:** Mengmeng Zhou, Xuanhao Fu, Boyuan Ma, Ziyu Chen, Yuelin Cheng, Linyan Liu, Shunli Kan, Xinyan Zhao, Sa Feng, Zehua Jiang, Rusen Zhu

**Affiliations:** Tianjin Union Medical Center, Tianjin, China

**Keywords:** low-intensity ultrasound, blood-brain barrier opening, Alzheimer's disease, β-amyloid, tau

## Abstract

Due to the complex pathological mechanisms of Alzheimer's disease (AD), its treatment remains a challenge. One of the major difficulties in treating AD is the difficulty for drugs to cross the blood–brain barrier (BBB). Low-intensity ultrasound (LIUS) is a novel type of ultrasound with neuromodulation function. It has been widely reported that LIUS combined with intravenous injection of microbubbles (MB) can effectively, safely, and reversibly open the BBB to achieve non-invasive targeted drug delivery. However, many studies have reported that LIUS combined with MB-mediated BBB opening (LIUS + MB-BBBO) can improve pathological deposition and cognitive impairment in AD patients and mice without delivering additional drugs. This article reviews the relevant research studies on LIUS + MB-BBBO in the treatment of AD, analyzes its potential mechanisms, and summarizes relevant ultrasound parameters.

## 1. Introduction

Treating neurological and psychiatric disorders remains a challenge due to their complex pathogenesis, resulting in significant economic burdens on both patients and society. Taking Alzheimer's disease (AD) as an example, it is the most prevalent neurodegenerative disease, and its pathogenesis has not been fully elucidated (1). In pathology, AD is characterized by the accumulation of intracellular hyperphosphorylated tau protein and the deposition of extracellular β-amyloid (Aβ) plaques (2). One of the major challenges in treating AD is the difficulty of drugs crossing the blood–brain barrier (BBB), which results in low-drug bioavailability (3). Hence, various methods have been developed to bypass the BBB to directly access the brain, such as intraventricular injection. However, these approaches often come with numerous adverse effects (4, 5).

Over the past 2 decades, researchers have found that low-intensity ultrasound (LIUS) combined with intravascular microbubbles (MB) injection can safely and temporarily open the BBB (6), enhancing the efficacy of therapeutic antibodies (7–10). Interestingly, a growing number of studies have shown that LIUS combined with microbubble-mediated BBB opening (LIUS + MB-BBBO) without drug delivery can improve pathology and cognitive impairment in AD patients and mice (11–14). This is undoubtedly a remarkable discovery.

In this article, we review relevant preclinical and clinical studies on LIUS + MB-BBBO in improving AD. We have discussed the possible mechanisms underlying this effect and summarized the relevant parameters.

## 2. The impact of the BBB on AD

The BBB is a specialized structure located between blood vessels and the brain tissue. It is mainly composed of vascular endothelial cells and surrounding glial cells and plays a vital role in protecting and regulating the internal environment of the brain (15). The BBB, serving as a dynamic interface between the neural tissue and blood circulation, forms the anatomical foundation of the functional brain vascular unit (16) (17). Extensive research indicates a close association between BBB dysfunction and AD. Many scholars consider this dysfunction to be an early hallmark of AD (3). The BBB dysfunction leads to the accumulation of harmful substances in the brain and impaired clearance, subsequently triggering immune responses and inflammation, which accelerate neurodegeneration. For instance, a significant portion of Aβ plaques is primarily cleared from the brain through various transport proteins located at the BBB. Due to the BBB dysfunction, the expression of these proteins such as low-density lipoprotein receptor (Aβ clearance receptor) (18), APOE isoform-related receptors (19), and P-glycoprotein (20) is reduced, leading to accelerated accumulation of Aβ plaques in the brain (21). Furthermore, the correlation between BBB dysfunction and tau pathology has been extensively reported (22). Hence, considering the BBB as one of the strategies for treating AD may hold a potential value.

## 3. LIUS combined with microbubbles can effectively open the BBB

In general, LIUS refers to ultrasound waves with an intensity level similar to or lower than those commonly used in diagnostic examinations in the United States (23), and many articles describe it as low-intensity pulsed ultrasound (LIPUS) (24, 25). LIUS primarily consists of periodic mechanical sound waves that propagate through cells and tissues, generating vibrations and collisions, resulting in beneficial biological effects and minimal thermal impact (26). LIUS demonstrates positive effects in neuromodulation, including enhancing neuronal activity (27), inhibiting neural inflammation (28), and stimulating the potential for neural differentiation in stem cells (29).

Over the past decade, with the development of ultrasound transducers, the introduction of low-intensity focused ultrasound (LIFU) has improved traditional pulse ultrasound treatment methods. Common LIFU ultrasound modes include MRI-guided focused ultrasound surgery (MRgFUS) (11) and scanning ultrasound (SUS) (10, 30). Unlike high-intensity focused ultrasound, LIFU has the capability to precisely converge energy with varying intensities and frequencies onto specific focal points in the brain, allowing for non-invasive treatment of central nervous system disorders. Combined with the intravascular MB injection, LIFU can effectively, safely, and reversibly open the BBB (31, 32).

**Figure 1:**
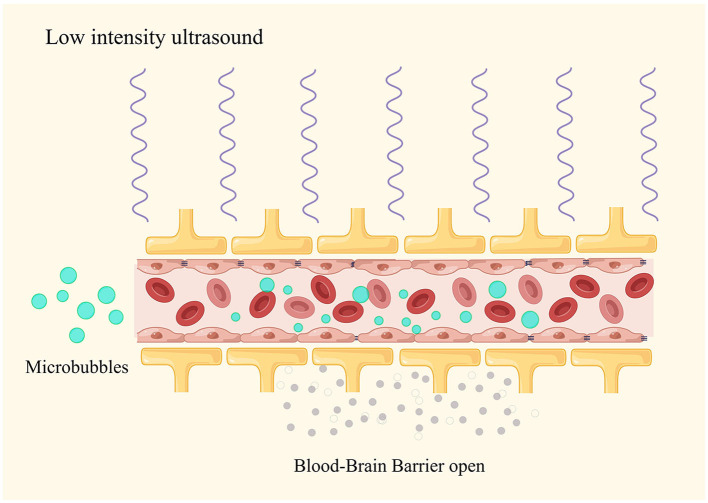
LIUS combined with microbubbles to open the blood–brain barrier.

In ultrasound therapy, the acoustic parameters (sound pressure, frequency, and pulse duration) as well as the size and concentration of MB determine the size of the BBB opening (33, 34). When applying ultrasound stimulation to MB (sized from 1 to 10 micrometers), the expansion and contraction of the bubbles result in mechanical stretching of the blood vessel walls. This mechanical stretching can alter the characteristics of endothelial cells, increasing their phagocytic activity and creating intercellular gaps in the endothelium (35) (Figure 1). Furthermore, ultrasound stimulation can open tight junctions, especially proteins such as occludin and claudin-5 (36). BBB opening is transient, and the integrity can rapidly recover within 6–24 h following the ultrasound treatment (37). In addition to changes in the physical barrier, ultrasound in combination with MB can also influence the function of the BBB. Within 48 h after the ultrasound treatment, there is a decrease in P-glycoprotein expression, but endothelial cell function remains unimpaired. These mechanisms gradually appear in two distinct phases of BBB opening: an early/fast leak and a late/slow leak (38). These findings confirm the impact of ultrasound on the BBB efflux mechanisms (39).

## 4. Biological effects of LIUS + MB-BBBO on AD

Preclinical studies have extensively validated the safety and feasibility of LIUS + MB-BBBO in various species, including small rodents and non-human primates (40). This strategy achieves spatial and temporal targeted non-invasive brain drug delivery (41, 42). Research has reported that LIUS + MB-BBBO has the ability to clear Aβ plaques without the need for additional drug administration (10, 43, 44). Jessica employed LIFU combined with MB-mediated BBB opening (LIFU + MB-BBBO) to deliver low-dose Aβ antibodies to reduce the pathological characteristics of Aβ plaques (45). In 2013, she first discovered the potential effects of ultrasound: under the same parameters and without the supplementation of exogenous antibodies, LIFU + MB-BBBO helps to reduce the area of Aβ plaques. LIFU + MB-BBBO allows endogenous immunoglobulins to enter the brain to help plaque clearance, where IgM levels are positively correlated with BBB opening. Additionally, LIFU treatment activates glial cells located near the plaques within the cortex and enhances the internalization and phagocytosis of Aβ plaques within these cells (9).

Neuroglial cells likely play a role in the process of Aβ plaques clearance, with microglial cells responsible for breaking down larger plaques into smaller components and absorbing them. However, this effect may have its limitations (46). Compared to peripheral immune cells, microglial cells have a weaker phagocytic function (47). Studies have reported that LIFU + MB-BBBO can induce local blood-borne mononuclear cell infiltration (13) (48). Charissa (13) believed that the peripheral immune response triggered by LIFU + MB-BBBO can effectively clear Aβ plaques. LIFU + MB-BBBO induces local recruitment of neutrophils, which can help Aβ plaques clearance by recruiting other downstream immune cells, such as monocytes and macrophages with stronger clearance capabilities than neutrophils (49). In addition, Gerhard (10) believed that SUS combined with MB-mediated BBB opening (SUS + MB-BBBO) can promote blood-borne immune molecules and albumin to enter the brain, and then albumin can assist glial cells and perivascular macrophages to engulf Aβ plaques (50). The above results indicate that endogenous immune upregulation triggered by LIUS + MB-BBBO is a possible mechanism for Aβ plaque clearance.

Tau is a protein associated with microtubules. Phosphorylated tau causes neuronal dysfunction and the formation of neurofibrillary tangles. The accumulation of phosphorylated tau is a key pathological feature of AD (51). SUS + MB-BBBO can reduce the deposition of tau protein in the hippocampus and improve the memory function of K369I tau transgenic mice. Interestingly, the researchers found that this effect was through activation of autophagy in neurons, rather than through glial cell clearance of tau (52). The neuronal autophagy process is impaired in AD (53), so this may be a potential mechanism. The immune upregulation stimulated by LIFU + MB-BBBO is not unsuitable for tau pathology. Maria (54) found that LIFU + MB-BBBO reduced bilateral hippocampal p-tau in the rTg4510 mouse model and was accompanied by an upregulation of immune responses. The researchers observed microglia colocalizing with phosphorylated tau. In addition, Amandine (55) explored the effect of LIPUS on tau transgenic P301S mice. The study found that LIPUS + MB-BBBO does not reduce tau pathology and may even aggravate the accumulation of pathological tau in stimulated brain areas. LIPUS combined with MB-mediated BBB opening (LIPUS + MB-BBBO) strongly reduced microglia density in the brain parenchyma of P301S mice and exhibited anti-inflammatory effects. This suggests that reduced microglial load may impair phagocytosis of tau and other aggregated debris, thereby limiting steady-state tau clearance.

Other studies have shown that MRgFUS combined with MB-mediated BBB opening (MRgFUS + MB-BBBO) can cause cerebrospinal fluid extravasation in AD patients, which is related to lymphatic efflux and enhanced meningeal venous permeability (56, 57). Convection in the cerebrospinal fluid of higher mammals rapidly removes proteins, and particles with larger molecular weights are removed through diffusion (58). This effect may be related to the clearance of Aβ plaques. In summary, LIUS + MB-BBBO can activate autoimmunity, autophagy, and lymphatic efflux to promote the clearance of pathological deposits, which can help improve cognitive function, but further research is needed to elucidate the biological effects of this pathway.

## 5. Related parameters of LIUS + MB-BBBO

Due to anatomical differences in the brain, skull, and cerebral vasculature between small and large animals, the parameters for LIUS + MB-BBBO have species-specific differences (59). This review summarizes relevant parameters of clinical studies on LIUS + MB-BBBO (Table 1).

**Table 1:** Clinical trials on low-intensity ultrasound combined with microbubbles to open the blood-brain barrier.

**Researcher/year**	**Ultrasound type**	**Treatment area**	**Parameter**	**Research object**	**Sample size**	**Treatment cycle**	**Adverse event**	**Last follow-up time**
Lipsman et al. (11)	MRgFUS	Right frontal lobe	220kHz	Early AD patients	5	2 stages, 1 month apart	None	2 months after treatment
Rezai et al. (12)	MRgFUS	Hippocampus and entorhinal cortex	220kHz	Early AD patients	6	3 stages, 2 weeks apart	None	1 month after treatment
Epelbaum et al. (8)	LIPUS implantable device	Left pariotemporal junction	1 MHz	Early AD patients	10	7 stages, 2 weeks apart	None	8 months after treatment
Park et al. (14)	MRgFUS	Bilateral frontal lobe	220kHz	AD patients	5	2 stages, 3months apart	None	3 months after treatment
D'Haese et al. (7)	MRgFUS	Hippocampus and entorhinal cortex	220 kHz	Early AD patients	6	3 stages, 2 weeks apart	None	90 days after treatment
Mehta et al. (56)	MRgFUS	Frontal, parietal and medial temporal lobes	220 kHz	Early AD patients	8	3 stages, 2 weeks apart	None	36 weeks after treatment
Rezai et al. (60)	MRgFUS	Hippocampus, frontal and parietal lobes	220 kHz	Early AD patients	10	3 stages, 2 weeks apart	None	6-12 months after treatment

MRgFUS, MRI-guided Focused Ultrasound Surgery; AD, Alzheimer's disease; LIPUS, low intensity pulsed ultrasound.

In Lipsman et al. (11) used MRgFUS at a frequency of 220 kHz to open the right prefrontal BBB in a clinical study of five AD patients for the first time. The ultrasound coverage consisted of a 3 by 3 grid of spots, each 3 mm in diameter, with the axial size of the target adjusted according to the subject degree of brain atrophy. Ultrasound was applied to each grid for a total of 50 s, and each point was ultrasonically treated with 2 ms on and 28 ms off burst pulses for 300 ms, the repetition interval was 2.7 s (duty cycle 0.74%). After 1 month, the BBB was opened at the original location and adjacent areas according to the same parameters. The tissue volume opened this time was twice that of the previous time. This study demonstrates the clinical safety and technical feasibility of MRgFUS + MB-BBBO. Based on this study, Rezai et al. (12) conducted a multicenter phase II trial in six AD patients, demonstrating that MRgFUS can safely, reversibly, and accurately open the BBB located in deep brain structures (hippocampus and entorhinal cortex) (opening degree: 95%). In this study, the researchers selected five targets of 5 × 5 × 7 mm3, and the opened hippocampal BBB was ~2–3 cm3. However, due to limited sample size, neither study observed changes in patients' cognitive function or reduction in Aβ plaque deposition. Rezai et al. (60) increased the number of subjects (10 AD patients) in subsequent clinical studies and used MRgFUS to open the BBB in multiple brain locations (hippocampus, frontal lobe, and parietal lobe). On follow-up for 6–12 months after ultrasound treatment, the researchers observed that compared with before treatment, amyloid levels in the FUS target area decreased by an average of 5%, and the patients' Centiloid scale decreased by 14%, without serious related adverse events.

In addition, there are some exciting results. D'Haese et al. (7) used MRgFUS + MB-BBBO in a clinical trial of six AD patients. At 7 days of follow-up after treatment, the researchers observed a slight decrease in amyloid levels compared with before treatment, with an average decrease of 5% in patients, but no cognitive assessment was observed. Park et al. (14) conducted a clinical trial of MRgFUS + MB-BBBO, the average volume targeted by FUS was 21.1 ± 2.7 cm^3^. Five AD patients received two treatments every 3 months. The results showed that MRgFUS + MB-BBBO reduced Aβ deposition (−1.6%) and improved the patients' neuropsychiatric symptoms.

In addition to the above-mentioned extracranial devices, implantable devices have also entered the clinical stage (61, 62). Epelbaum (8) implanted an implantable LIPUS device (1-MHz) into the skulls of 10 patients with mild AD under local anesthesia, and this device had previously been used in a Phase I/IIa study of patients with recurrent glioblastoma (61). The results showed that the implantable LIPUS device combined with MB was effective and safe in opening the BBB, and in all cases, the researchers observed a reduction in amyloid burden after 4 months of follow-up. Due to the small sample size and short follow-up period, no cognitive changes were detected.

## 6. Outlook and challenges

Overall, there have been several small cohort clinical trials demonstrating the safety and feasibility of LIUS + MB-BBBO in AD patients (Table 1). However, due to the small sample size and short follow-up time of these studies, whether LIUS + MB-BBBO can reduce amyloid burden and improve cognition in AD patients still needs to be confirmed by more large cohort studies. In addition, the complete pathophysiology of AD is still unclear. Even though several preclinical studies have tried to reveal the mechanism of LIUS + MB-BBBO on AD, there are few relevant reports. Therefore, the main goals in future are to improve our understanding of the pathophysiology of AD and further explore the mechanism and maximum energy efficiency of ultrasound-mediated BBB opening in improving AD. Furthermore, the neuromodulation effects of LIUS cannot be ignored, including increasing the activity of neurons (27), promoting the release of neurotrophic factors in the brain (63), and promoting the neural differentiation potential of stem cells (29), non-invasive drug delivery (7–10), etc. It is necessary to consider these effects together to provide new opportunities for improving the treatment of neurodegenerative diseases.

## 7. Conclusion

LIUS + MB-BBBO may be a potential treatment for AD.

## Author contributions

MZ: Conceptualization, Investigation, Writing—original draft, Writing—review and editing. XF: Visualization, Writing—original draft, Writing—review and editing. BM: Investigation, Writing—original draft. ZC: Writing—review and editing. YC: Data curation, Methodology, Writing—review and editing. LL: Methodology, Writing—original draft. SK: Funding acquisition, Writing—review and editing. XZ: Data curation, Methodology, Writing—review and editing. SF: Data curation, Writing—review and editing. ZJ: Data curation, Writing—review and editing. RZ: Funding acquisition, Resources, Writing—review and editing.
